# Italian public’s views on sharing genetic information and medical information: findings from the ‘Your DNA, Your Say’ study

**DOI:** 10.12688/wellcomeopenres.16909.1

**Published:** 2021-07-12

**Authors:** Virginia Romano, Richard Milne, Deborah Mascalzoni

**Affiliations:** 1 Center for Research, Ethics and Bioethics, Uppsala University, Uppsala, Sweden, SE-751 05, Sweden; 2 Medical Ethics, Lund University, Lund, Sweden, 22362, Sweden; 3 Department of Public Health and Primary Care, University of Cambridge, Cambridge, UK, UK; 4 Society and Ethics Research, Wellcome Connecting Science, Wellcome Genome Campus, Hinxton, UK, CB 10 1SA, UK; 5 Institute of Biomedicine, Eurac Research, Bolzano, Italy, 39100, Italy

**Keywords:** DNA sharing, biobanks, bioethics, public attitudes, data sharing, Italy

## Abstract

**Background**: The collection and sharing of genomic and health data underpins global efforts to develop genomic medicine services. ‘Your DNA, Your Say’ is a cross-sectional survey with the goal of gathering lay public attitudes toward the access and sharing of deoxyribonucleic acid (DNA) information and medical information. It suggests significant international variation in the willingness to share information, and in trust in the actors associated with the collection and use of this information. This paper explores these questions in the Italian context.

**Methods**: The Italian Your DNA, Your Say campaign led to the collection of 1229 valid questionnaires. The sample was analysed using standard descriptive statistics. We described the sample in terms of gender, age ranges and self-reported religiosity, and split the sample amongst the five typically studied Italian macro-areas to explore regional variation. We analysed the relationship between these factors and trust and willingness to share medical and DNA information.

**Results**: The majority of the sample, across all socio-demographics, were willing to share DNA and health information with all entities considered except for-profit researchers. Respondents tended not to trust institutions beyond their own doctor. There was no difference between Italian regions.

**Conclusions**: Despite the generally positive attitude towards sharing, we suggest that the lack of trust in non-profit researchers and the government needs to be better understood to inform public communication projects around genomics in the future and to enhance awareness of DNA and medical information in Italy.

## Introduction

In order to achieve its potential to predict, diagnose, manage and treat genetic disease ( Middleton *et al.*, 2018), clinicians and researchers working in genomic medicine need access to data from a diverse range of people across multiple datasets ( Stark *et al.*, 2019). Public and patient support for and trust in the collection and sharing of genomic data is thus critical to realizing the potential of genomic medicine, making it imperative to understand the possible reasons against sharing and how to most effectively address these. It is therefore essential to both raise awareness of genomics among the general public and to better understand what issues, doubts and resistance the general public might have towards privacy issues ( Kaye, 2012) and the responsible sharing and use of genomic information ( Knoppers, 2014; Kosseim *et al.*, 2014; Phillips *et al.*, 2020). Empirical research examining public attitudes, values and beliefs is particularly relevant to give voice to those who are or will be directly affected by genomics ( Middleton *et al.*, 2018), and because they allow us to explore the characteristics of those who are unwilling to share and their reasons ( Middleton *et al.*, 2019). This is important in ensuring trust in genomic data sharing ( Milne *et al.*, 2019).

The overall findings of the Your DNA, Your Say (YDYS) study suggest significant diversity between countries in the willingness to share DNA (or genetic) information, in trust in the different actors responsible for collecting and using DNA and health data, and in public familiarity with genomics ( Middleton *et al.*, 2020). However, these overall patterns tell us little about the specific national, cultural, scientific and clinical contexts in which DNA and health data are collected, shared and used. In this paper we examine the Your DNA, Your Say data from Italy to consider in detail attitudes towards the sharing of genomics and health data in Italy.

Significant investment has been made by the Italian government in genomic data initiatives; the Italian Ministry of Health introduced a strategic policy plan on genomics and predictive medicine in 2010 ( Mazzucco *et al.*, 2017), and Italy is among signatories to the European 1+ Million Genomes initiative (MEGA). However, research exploring familiarity and attitudes of Italian clinicians and health professionals towards genomic medicine, has suggested a need for additional clinical education ( Boccia *et al.*, 2014; Marzuillo *et al.*, 2014). As yet however, there is little evidence on public attitudes towards the sharing of health and genomic data in the Italian context and the plans and activities of the Italian Ministry of Health in this area are far from being widely familiar to the general public ( Simone *et al.*, 2013). A previous comparative analysis of the Your DNA, Your Say data ( Middleton *et al.*, 2020), suggests that around 40% of Italians are familiar with DNA, genetics or genomics. In this paper, we draw on the Your DNA, Your Say data to examine how attitudes towards sharing vary across socio-demographic groups and what data about Italian respondents tells us about trust in this national context. In addition, we develop an exploratory analysis of variation between Italian regions in terms of the willingness to share and trust in actors responsible for collecting, sharing and storing genomic data.

## Methods

The study is based on the Italian translation of the cross-sectional ‘Your DNA, Your Say’ survey that aims to gather lay public attitudes toward the sharing, access and sharing of DNA information and medical information ( Middleton *et al.*, 2018). Data were collected using an online survey containing 29 questions presented in one of 22 languages – in this case, in Italian. The survey uses short video clips, presented in English and subtitled in Italian, to explain in lay terms the main reasons for and implications of the sharing and uses of DNA and medical information; moreover, it describes the different subjects to whom it is possible to share, the different purposes for which shared data may be used, and considers the ethical issues surrounding this decision. An English version of the survey can be found as extended data ( Middleton *et al.*, 2021).

The Italian campaign was conducted through the market research company ResearchNow (now Dynata), and resulted in the collection of 1,229 valid questionnaires. The survey was designed to take 15–20 minutes to complete ( Middleton *et al.*, 2018). Recruitment aimed at obtaining a sample that was as representative as possible of the Italian population with regard to gender, age, and education level. Italian data were downloaded on 27 August, 2019. Data were downloaded by country and then merged. The following initial cleaning rules were applied to the full data set (regardless of participant country):

- Removal of incomplete responses
- Removal of surveys which took five minutes or less to complete
- Removal of surveys in which the word 'test' (or some variant thereof) was included in free-text fields

### Statistical analysis

The Italian sample was analysed using standard descriptive statistics and chi-squared tests using IBM SPSS Statistics version 27. The data can also be analysed using the open-source package R. We started with an accurate description of the sample characteristics in terms of gender, age range and self-reported religiosity. Age was collected in ten-year categories from 16 onwards. Due to fewer responses in younger and older categories these were collapsed into categories of “30 years and under”, “31–40”,”41–50”, ”51–60“, and “61 years and older'' for analysis. Gender was self-described “Female” or “Male”. Whether participants had children was determined by a “Yes'' or “No'' answer. Level of education was categorized as “Tertiary'', “Secondary'', “Primary'' or “Other'' based on structured responses and free-text descriptions of respondents' highest level of educational attainment. This was collapsed to a binary indicator of tertiary education. The exact age ranges associated with education categories vary between countries. However, we were interested in the highest level of educational attainment – particularly the difference between school and university education.

Religiosity was established based on responses to a question which asked “Whether you attend religious services or not, would you say you are … ?'' with options “A religious person'' or “Not a religious person''.

To explore regional variation, data were further split to capture the geographical distribution amongst the five typically studied Italian macro-areas, based on manual coding of participants’ responses to the question “Where do you live?”. Here we follow the Italian statistical standard whereby Italy is officially divided into five macro areas: Northwest (Piedmont, Aosta Valley, Liguria, Lombardia), Northeast (Trentino-Alto Adige, Veneto, Friuli-Venezia Giulia, Emilia-Romagna), Center (Toscana, Umbria, Marche, Lazio), South (Abruzzo, Molise, Campania, Puglia, Basilicata, Calabria) and Islands (Sicilia, Sardegna). Differences were explored using descriptive statistics and chi-square tests. Because the sample was not collected with reference to these regions, these sub-groups are necessarily small and less representative of regional populations than the overall sample is of the Italian population. However, given the potential importance of regional variation discussed above, this analysis may offer significant value and avenues for future exploration.

We then analysed the relationship between these descriptive variables and two main dependent variables using chi-square tests: trust towards different entities and willingness to share medical and DNA information to the same mentioned entities. 

### Ethics and consent

The online survey was fully anonymous. Participants were informed that their consent is given when they choose to click off the landing page and start answering the questions. On the landing page, the purpose of the project is explained as well as what participation involves, participants have a choice at any stage within the survey, to stop answering the questions and withdraw. The online project is physically based at the Wellcome Genome Campus with all data collected and stored in encrypted files at the Wellcome Sanger Institute in Cambridge. As part of the conditions of research delivery at this research institution the project passed ethical review by the Human Materials and Data Management Committee of the Wellcome Sanger Institute (Registration Number: 16/029) as well as legal review to ensure that it was compliant with ethical and legal standards for participant involvement, data collection and storage.

## Results

The socio-demographic distribution of the sample is described in Table 1 ( Middleton *et al.*, 2021). The largest group of respondents (301, 24.5%) was in the age range between 41 and 50 years, and the sample was balanced between males and females (627:602). The majority of the sample (846, 68.8%) obtained secondary education, while 64% (786) self-reported being “a religious person”.

**Table 1.** Sociodemographics of Your DNA, Your Say (YDYS) Italy sample.

Variable	Categories	Italy	
		N	%
Age category (years)	30 and under	218	17.7
	31–40	215	17.5
	41–50	301	24.5
	51–60	237	19.3
	Over 60	258	21
Gender	Female	627	51
	Male	602	49
Has children	No	458	37.3
	Yes	761	61.9
	Missing	10	0.8
Highest education level	Tertiary	351	28.6
	Secondary	846	68.8
	Primary	18	1.5
	Other	13	1.1
	Missing	1	0.1
Religiosity	Not a religious person	443	36
	A religious person	786	64
	Missing	0	0

In terms of the geographic distribution of the sample ( Table 2), 334 (27.2%) respondents did not provide a response to this question that enabled their region to be classified, and as such are excluded from the regional analysis. Of those included, the majority of the sample (35.4%) replied from northwest regions, while only 10.2% of respondents came from the two main Italian islands. As can be seen, while the distribution of respondents in the two northern regions differs from the overall Italian population, the split between North, Central, South and Islands approximates that of the Istat (2019) data.

**Table 2.** Geographic distribution of the Your DNA, Your Say (YDYS) Italy sample, compared to official population data.

Macro-area	Frequency	Percentage of sample (excluding missing)	Percentage of Italian population (iStat data)
Northwest	317	35.4%	26.8%
North East	112	12.5%	23.0%
Center	178	19.9%	19.8%
South	197	22.0%	19.5%
Islands	91	10.2%	10.9%
Total	895	100%	100%

### Willingness to share

Overall, willingness to share is high amongst our sample ( Table 3). Most (64%) declared they would be willing to share their DNA and medical information for use by at least one data user, while those who were unwilling (14%) and the undecided (22%) represent a minority of the sample as a whole.

**Table 3.** Variation in willingness to share medical and deoxyribonucleic acid (DNA) information by actor, demographics, religiousness, region and general trust.

		Willingness to share with at least 1			Share with Doctor			Share with non-profit user			Share with for profit user		
		No	Unsure	Yes	No	Unsure	Yes	No	Unsure	Yes	No	Unsure	Yes
		Row N %	Row N %	Row N %	Row N %	Row N %	Row N %	Row N %	Row N %	Row N %	Row N %	Row N %	Row N %
Age (years)	30 and under	13.8%	11.0%	75.2%	8.8%	25.8%	65.4%	13.3%	30.3%	56.4%	23.9%	39.0%	37.2%
	31–40	16.7%	8.8%	74.4%	9.3%	26.5%	64.2%	12.1%	25.6%	62.3%	25.7%	33.2%	41.1%
	41–50	21.6%	15.3%	63.1%	12.0%	31.6%	56.5%	14.3%	31.6%	54.2%	26.9%	33.9%	39.2%
	51–60	24.9%	18.6%	56.5%	14.8%	24.1%	61.2%	14.3%	25.3%	60.3%	29.1%	30.8%	40.1%
	Over 60	30.6%	16.3%	53.1%	14.7%	29.8%	55.4%	17.4%	27.9%	54.7%	27.5%	38.0%	34.5%
Gender	Male	18.6%	14.3%	67.1%	11.8%	25.8%	62.4%	13.8%	25.6%	60.6%	26.4%	34.6%	39.0%
	Female	25.0%	14.2%	60.8%	12.3%	29.8%	57.9%	15.0%	30.9%	54.1%	27.0%	35.3%	37.7%
Tertiary education	No	23.8%	17.0%	59.2%	12.5%	29.6%	57.8%	14.7%	30.0%	55.4%	26.5%	36.3%	37.3%
	Yes	17.1%	7.4%	75.5%	10.8%	23.4%	65.8%	13.7%	24.2%	62.1%	27.4%	31.6%	41.0%
Macro area	North West	24.6%	15.8%	59.6%	13.9%	29.0%	57.1%	14.8%	30.9%	54.3%	24.3%	35.0%	40.7%
	North East	16.1%	13.4%	70.5%	7.1%	21.4%	71.4%	9.8%	30.4%	59.8%	25.9%	37.5%	36.6%
	Central	19.1%	12.9%	68.0%	10.1%	29.2%	60.7%	12.4%	28.1%	59.6%	29.8%	32.0%	38.2%
	South	23.9%	15.7%	60.4%	10.7%	33.2%	56.1%	14.7%	31.5%	53.8%	27.0%	36.2%	36.7%
	Islands	20.9%	13.2%	65.9%	13.2%	18.7%	68.1%	12.1%	24.2%	63.7%	20.9%	38.5%	40.7%
Religion	A religious person	23.3%	16.0%	60.7%	12.7%	28.5%	58.7%	14.2%	29.1%	56.6%	24.7%	33.8%	41.5%
	Not a religious person	19.4%	11.1%	69.5%	10.8%	26.6%	62.5%	14.7%	26.9%	58.5%	30.2%	37.0%	32.7%
Trust	No	29.0%	17.2%	53.8%	16.5%	35.8%	47.7%	19.5%	36.4%	44.2%	33.9%	38.8%	27.3%
	Yes	11.0%	9.7%	79.3%	5.2%	15.7%	79.1%	6.6%	15.9%	77.5%	15.7%	28.9%	55.4%

**Figure 1.**
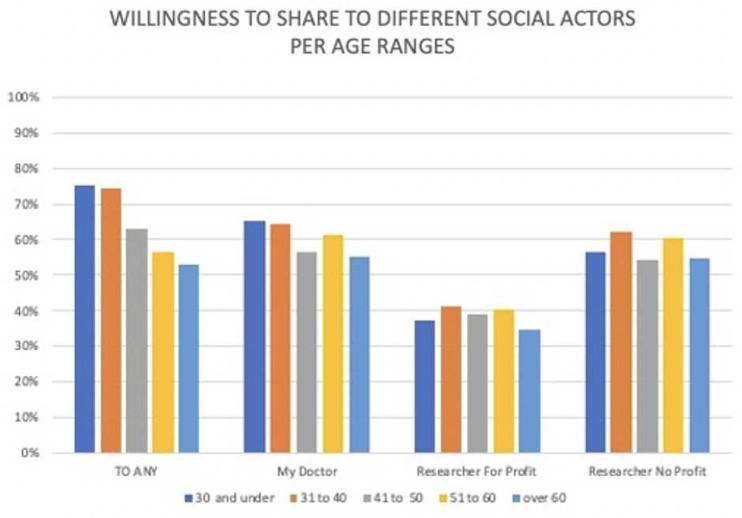
Willingness to share medical and deoxyribonucleic acid (DNA) data with different social actors by age group.

***Age.*** Overall, positive attitudes towards sharing – i.e. those who were willing to share data with at least one actor – were significantly higher for those 30 years and under (75.2%) and 31 to 40 years (74.4%) than other groups, declining substantially for the over 60s (53.1%, p<0.001) but differences for specific recipients of data are not significant (see Figure 1 and Table 3). The overall attitude towards sharing is positive (60% for sharing with their doctor and 57% for sharing with non-profit researchers).

When asked about willingness to share with for-profit researchers, the overall percentage of uncertain respondents is higher (35%) —and for this category of recipient only— is higher than that of negative respondents (27%).

**Figure 2.**
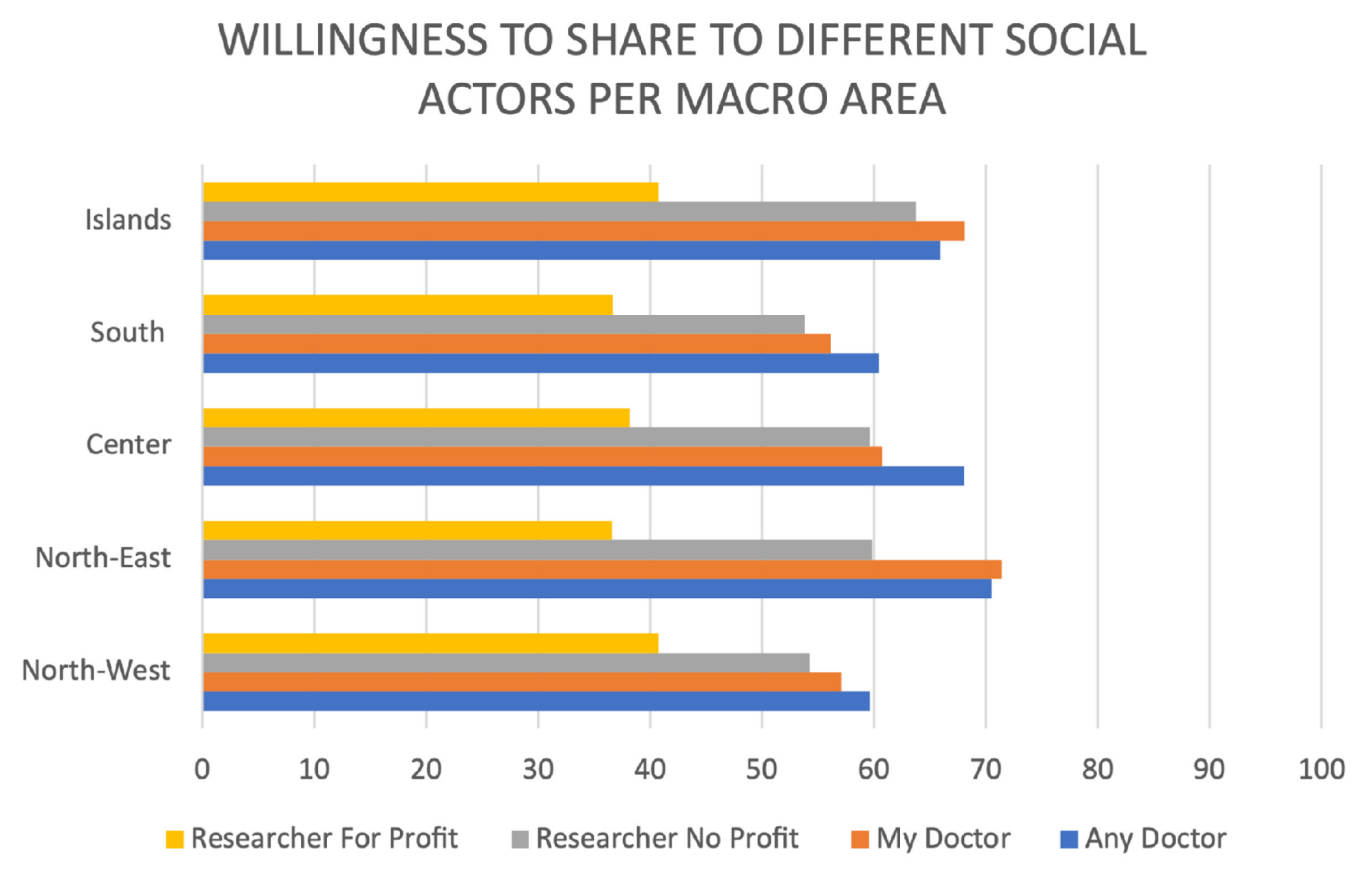
Willingness to share medical and deoxyribonucleic acid (DNA) data by actor and macro area.

***Regional analysis.*** The general attitude towards willingness to share seems stronger both in Northern regions (59.6% and 70.5% positive respondents, respectively, for North West and North East) followed by Islands (see Figure 2), across all categories of recipient social actors. Southern regions are less inclined for sharing (with a general positive attitude towards sharing of 60.4%), while central regions have a halfway position (68% of positive respondents to sharing in general). Although these variations are interesting, overall differences were not statistically significant.

**Figure 3.**
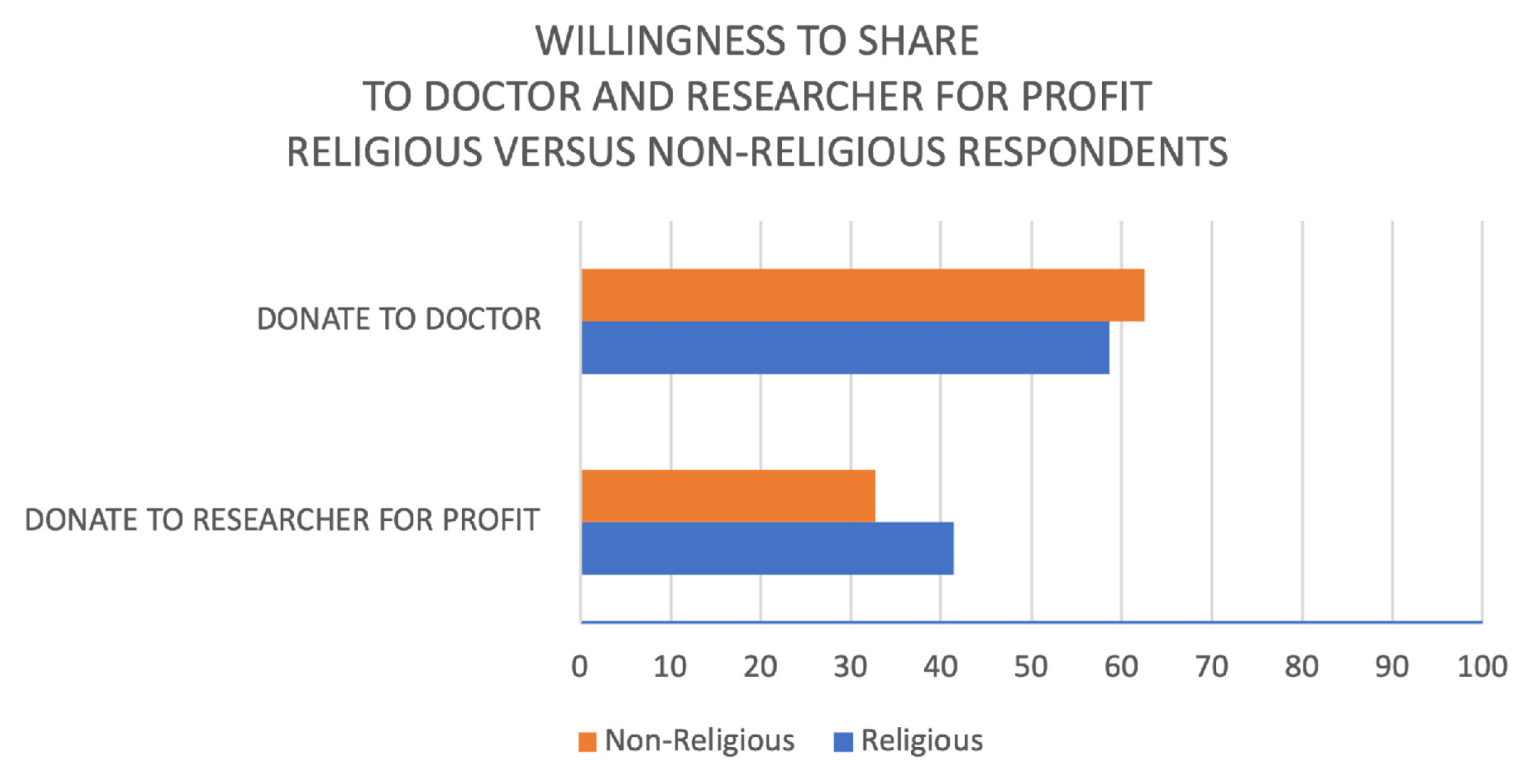
Willingness to share medical and deoxyribonucleic acid (DNA) data to doctor or researcher for profit by religion.

***Religiosity.*** Overall, non-religious respondents were significantly more likely to be willing to share with at least one data user than religious respondents (69.5 vs 60.5%, p=0.006, see Table 2). However, this varied substantially between data users. Both religious and non-religious respondents expressed a positive attitude towards sharing with a peak of 62.5% of non-religious persons to their doctor. This general trend has one main exception regarding attitudes towards for-profit researchers: in this case, the percentage decreases to 34% and merges into the “uncertain” category (see Figure 3).

Overall, religious participants appear more cautious in terms of their attitude towards sharing. However, in terms of willingness to share to for-profit researchers, there is a significantly higher positive response among those who describe themselves as religious (41.5%) than among non-religious people (32.7%; p=0.05). However, the distance between positive attitude between religious and non-religious respondents does not appear as significant as the distance between attitudes towards the recipients of sharing.

***Trust.*** Overall, those people with the highest level of trust – i.e. those who state that they trust two or more actors – are significantly more likely to be willing to share. In the following section, we consider who is most likely to trust, and how this varies across the Italian sample.

***Age.*** The level of trust varies by actor, but is low for all categories except My Doctor, on average lower than 30% and as low as 8% when respondents are asked whether or not they trust their government ( Table 4). When the category changed to other doctors, social actors and institutions, stated trust dropped to a low of 8.6% (fFor-profit researcher) and a maximum of 36.7% (non-profit researcher). There was no significant difference in trust by age - the majority of people in all age ranges (71.4%) declared they trust their own doctors, varying from 77.1% (for people over 60) to a minimum of 66% (for people between 41 and 50 years).

**Table 4.** Variation in trust by actor, demographics and region.

		Trust in more than one actor		Trust in my doctor		Trust in any doctor in my country		Trust in non-profit researcher in my country		Trust in for-profit researcher in my country		Trust in the government of my country	
		No	Yes	No/ Not sure	Yes	No/ Not sure	Yes	No/ Not sure	Yes	No/ Not sure	Yes	No/ Not sure	Yes
Age	30 and under	58.3%	41.7%	27.5%	72.5%	73.9%	26.1%	65.1%	34.9%	86.7%	13.3%	88.5%	11.5%
	31–40	55.8%	44.2%	29.3%	70.7%	69.2%	30.8%	63.3%	36.7%	87.0%	13.0%	86.5%	13.5%
	41–50	65.1%	34.9%	33.2%	66.8%	79.4%	20.6%	71.1%	28.9%	91.4%	8.6%	91.7%	8.3%
	51–60	62.0%	38.0%	29.1%	70.9%	77.1%	22.9%	68.8%	31.2%	91.1%	8.9%	89.4%	10.6%
	Over 60	60.1%	39.9%	22.9%	77.1%	77.5%	22.5%	66.3%	33.7%	92.2%	7.8%	88.0%	12.0%
Gender	Male	59.0%	41.0%	28.7%	71.3%	75.9%	24.1%	65.1%	34.9%	89.5%	10.5%	86.9%	13.1%
	Female	62.2%	37.8%	28.4%	71.6%	75.7%	24.3%	69.2%	30.8%	90.3%	9.7%	91.1%	8.9%
Tertiary education	No	61.2%	38.8%	29.0%	71.0%	76.1%	23.9%	68.3%	31.7%	89.5%	10.5%	89.1%	10.9%
	Yes	59.3%	40.7%	27.4%	72.6%	74.9%	25.1%	64.4%	35.6%	90.9%	9.1%	88.9%	11.1%
Macro area	North West	83.2%	16.8%	24.3%	75.7%	74.1%	25.9%	65.3%	34.7%	89.3%	10.7%	90.2%	9.8%
	North East	81.3%	18.8%	25.0%	75.0%	72.3%	27.7%	64.3%	35.7%	89.3%	10.7%	90.2%	9.8%
	Central	85.4%	14.6%	29.8%	70.2%	76.4%	23.6%	63.5%	36.5%	88.8%	11.2%	91.0%	9.0%
	South	87.3%	12.7%	32.0%	68.0%	78.1%	21.9%	74.6%	25.4%	90.4%	9.6%	88.8%	11.2%
	Islands	83.5%	16.5%	23.1%	76.9%	79.1%	20.9%	67.0%	33.0%	87.9%	12.1%	79.1%	20.9%
Religion	A religious person	60.7%	39.3%	28.8%	71.2%	75.8%	24.2%	68.4%	31.6%	89.4%	10.6%	87.5%	12.5%
	Not a religious person	60.5%	39.5%	28.2%	71.8%	75.8%	24.2%	65.0%	35.0%	90.7%	9.3%	91.6%	8.4%

***Regional analysis.*** Levels of trust towards different subjects and institutions considered per macro regional area follow the same overall pattern, with the greatest trust in doctors, followed by non-profit researchers and finally for-profit research and government. Regions belonging to the islands consistently show the highest levels of self-reported trust i.e. towards their doctor (76.9% of positive respondents), followed by Northern regions (respectively scoring 75.7 % of positive respondents in north west and 75% in north east). Central regions (70.2%) come next, followed by southern regions (68%). Northern regions (both east and west) generally tend to have scores that are closer to islands than other macro areas (see Figure 2). However, these differences by region in the levels of trust in each actor are not significant (My doctor p=0.259; Country Doctor p=.673, Any doctor p=.624, non-profit p=.138, for profit, p=.977).

***Religiosity.*** No significant difference in trust towards different social actors was associated with religiosity (My doctor p=.842; Country Doctor p=.975, Any doctor p=.495; non-profit p=.218, for profit, p=.466).

## Discussion

In the Italian Your DNA, Your Say sample, overall willingness to share DNA and health information varies along key demographics. Specifically, older age, being female, having less than tertiary education, and expressing religious beliefs are associated with lower overall willingness to share. Overall trust (that is, trust in multiple data users) is also strongly associated with the willingness to share. However, no clear relationship can be identified between socio-demographic characteristics and either overall trust or trust in specific data users.

When considering overall willingness to share as a whole, we found similar attitudes towards doctors and non-profit researchers, suggesting that these actors are considered in a similar way. In contrast, when asked about willingness to share with for-profit researchers, the percentage of uncertain respondents was higher than that of negative respondents (Not sure 35%; No 27%). This strong uncertainty appears consistent with the general pattern of trust among our respondents.

Public trust in genomic data sharing is a complex matter ( Critchley *et al.*, 2015; Milne *et al.*, 2019) it depends both on personal attitudes as well as on the potential recipients and users of data, how their goals are perceived to be connected to the common good and how these matters are communicated to the wider lay public. Generally, Italian respondents reveal a low level of trust towards social actors such as doctors and institutions, with the major exception of the figure of their personal doctor. 

The distinctive attitudes towards personal doctor strengthens and consolidates the importance of trust as a key factor to understand willingness to share overall. Among the different potential recipients and users of data, the personal doctor is someone participants have known for a long time, who is seen as the main point of contact for their own health and who has had the chance to gain their trust in many different occasions ( Mechanic, 1998; Rowe & Calnan, 2006). As such, the personal doctor is the one actor participants are more likely to have a personal reason to trust. This pattern of responses also highlights the persistent importance of the family doctor in an Italian context, suggesting this professional figure could play a pivotal role in facilitating engagement about the sharing of DNA and medical information due to issues of access to and familiarity with patients. While more evidence is needed to clearly understand the role that this professional figure might play in the context of the different Italian regional health systems, our findings suggest that public health communication campaigns around genetic medicine could benefit from involving the family doctor to promote personalized forms of communication and facilitate recruitment.

Indeed, the data show low levels of trust in social actors that participants have little direct experience of, and whose interests and goals may be unknown. Our data suggests that trust in institutions or social entities perceived as distant and abstract cannot be taken for granted in the Italian context. The finding corroborates the overall pattern of responses found in the wider YDYS dataset as well as data specific to the Italian context ( Istat, 2019; Middleton *et al.*, 2020). Italian respondents show the highest levels of mistrust towards two specific categories: researchers for profit and their government. Trust towards government may be related to the historically high mistrust of Italian people towards institutions ( Hooghe & Stolle, 2003). However, the question of trust in for-profit researchers emphasizes the importance of motives when considering willingness to share data for use by different actors. In relation to for-profit research, previous studies have suggested that many people show a ‘natural prejudice’ ( Nicol *et al.*, 2016) against commercial use of health and genetic data. Issues surrounding the commercialization of health data, in general, and more specifically of genetic information are increasingly conceptualized as multi-faceted, recognizing the importance of bioethical and regulatory considerations related to the management of biobanks as well as their need to survive through some sort of compromise with the market ( Caulfield *et al.*, 2014; IPSOS Mori, 2016; McWhirter *et al.*, 2020). Nevertheless, even if commercialization of genetic data tends to generate a negative reaction, research in this field underscores how good communication and a better engagement of the public about the possible benefits for the common good deriving from commercial involvement ( Skovgaard *et al.*, 2019; Solum Steinsbekk *et al.*, 2013) could result in more positive public attitudes towards the involvement of for-profit actors. Economic and scientific interests are not extremes on a spectrum, and can be aligned and work together in the interest of the public. Effective science education and communication —before even considering questions surrounding DNA—might be a good starting point to be able to engage with complexity.

Privacy is a big issue in Italian culture ( Rodotà, 1995) especially if considered in the wider context of lack of trust towards institutions ( Hooghe & Stolle, 2003; Huysseune, 2003; Putnam *et al.*, 1993). We therefore feel confident in formulating the hypothesis that attitudes towards sharing is closely associated with issues of privacy and trust, and that these factors should be at the centre of further research on public views on the sharing of genomic data in Italy. It should also be noted that some of the strongest data we have on attitudes about willingness to share pertain to the percentages of uncertain respondents. Specifically, the number of people replying “I don’t know” to questions about their willingness to share medical and genetic information was consistently high, and often even higher than the number of people expressing positive or negative answers. In light of this finding, we suggest that this issue needs further investigation through both qualitative and quantitative methods, as this doubtful attitude could indicate a greater need for better communication about genetics and data sharing that is specifically targeted to the general public and to all age groups.

### Macro areas

Regional differences are potentially important in the Italian context for historical, statistical and administrative reasons ( Felice, 2011; Wagstaff, 1999), and in light of the regionalisation of the Italian health system ( Pavolini & Vicarelli, 2012; Toth, 2014; Vicarelli, 2015). However, the results presented do not allow to draw substantial conclusions about the differences between Italian regions, despite wider evidence of strong and persistent regional differences in people’s relationships towards institutions ( Putnam *et al.*, 1993). The lack of difference between regions may be partly due to the *post hoc* sampling and the high proportion of respondents who did not provide an accurate location within Italy. Nevertheless, the subtle variation we have started to observe suggests the potential value of further exploration building on historical and sociological work on cultural differences between Italian regions, particularly on differences in social and cultural capital ( Putnam *et al.*, 1993; Putnam, 2000).

### Religiosity

Allum and colleagues suggest that religion may act as a “perceptual filter”, moderating the relationship between knowledge and attitudes. ( Allum *et al.*, 2014: 846). Existing surveys on issues surrounding genomics, gene editing, gene therapy and bioethical issues associated with this technology have often identified (lower) religiosity as an explanation for a more positive attitude towards DNA sharing ( Pew Research, 2016; Sanderson, 2017). However, attitudes towards medical genetics are connected to religious beliefs in complex ways ( Allum *et al.*, 2014); a clear connection between religious affiliation and attitudes towards genetics is complicated by the need to consider a wider array of variables that may have a bigger impact on those very attitudes than religiosity (i.e. the severity of the condition for which genetic testing or similar tools are being accessed) ( Botoseneanu *et al.*, 2011).

In particular, an exception to this general association between (low) religiosity and willingness to share was evident in attitudes towards for-profit researchers. Though self-reported religious people appeared overall more cautious about sharing, this group was more willing than non-religious people to share data with for-profit researchers. More data is needed to fully understand the reasons behind these responses, particularly in light of the high percentage of uncertain responses. One hypothesis could be the potentially greater propensity of religious people to trust institutions. Against the general lack of trust in institutions that characterizes contemporary Italian society ( Hooghe & Stolle, 2003), religiosity may be a booster of social cohesion and, in a way, protect respondents from distrust. However, in our work, the difference in trust between self-reported religious and non-religious people is not significant, while the difference in willingness to share is, suggesting that on such a complex and multi-faceted issue such as DNA and medical information sharing, we do not yet have enough evidence to explain the role that religiosity plays and its interaction with factors such as familiarity with genetic disease (cf Porteri *et al.*, 2014). Future research on this theme might aim to understand better the specific influence of religiosity on public attitudes.

### Limitations

The overall limitations of the YDYS questionnaire have been reported elsewhere ( Middleton *et al.*, 2018). As an exploratory cross-sectional online survey, the study is limited in that it captures intended behaviour at a single time point. One specific limitation is the reliance in this analysis on self-reported data, particularly related to location and religiosity.

## Conclusions

The findings of the Your DNA, Your Say in Italy have value in starting to describe what the Italian population think about sharing genetic and health data, allowing an initial analysis of themes such as willingness to donate and trust in this specific sociocultural environment. As our data clearly show, given the average high occurrence of the answer “I don’t know” in any of the variables taken into consideration, there is still a great need to engage the Italian public with issues surrounding genomics, its clinical potential and risks. We believe that the findings presented in this article can be used to inform educational and engagement strategies and initiatives aimed at improving public awareness of genomics and data sharing in Italy. Specifically, the findings clearly point to two directions of future work: i) further research to better define a precise sociocultural profile of people positively and negatively oriented to both trust and sharing and ii) involvement of family doctors as facilitators.

## Data availability

### Underlying data

Open Science Framework: Your DNA Your Say data file. https://doi.org/10.17605/OSF.IO/ZPFGM ( Middleton *et al.*, 2021).

This project contains the following underlying data:

- YDYS dataset for sharing.csv

### Extended data

Open Science Framework: Your DNA Your Say data file. https://doi.org/10.17605/OSF.IO/ZPFGM ( Middleton *et al.*, 2021).

This project contains the following extended data:

- dataDictionary.csv
- pme-2018-0032.pdf (Description of study design)
- Word Version GA4GH Survey.docx

Data are available under the terms of the Creative Commons Attribution 4.0 International license (CC-BY 4.0).
