# Supplementary material for: Italian public’s views on sharing genetic information and medical information: findings from the ‘Your DNA, Your Say’ study
Source: Wellcome Open Res. 2021 Jul 12;6:180. [Version 1] doi: 10.12688/wellcomeopenres.16909.1 (PMC8855014; doi:10.12688/wellcomeopenres.16909.1)
Supplement: Peer review history

# Peer review: Reviewer response for version 1

**DOI:** 10.21956/wellcomeopenres.18654.r48305

**Published:** 2022-02-17

**Authors:** Hüseyin Demirci

**Affiliations:** 1 SnT, University of Luxembourg, Esch-sur-Alzette, Luxembourg

In this paper, the authors have analyzed the data from an Italian group of 'Your DNA, Your Say project' participants. While genome sequencing and direct-to-consumer services are becoming more available, this kind of study is crucial to better understand the privacy expectations of users. 

 The authors have analyzed the acceptance of genomics sharing against variables such as age, gender, residence location, education level, and religiosity. They analyzed the willingness to share with their special doctor, any doctor, for-profit and non-profit research purposes. The paper is generally well written and easy to read. The data analysis from the national and regional points of view gives important insights.

 I just have a minor comment that for instance Figure 1 has low image quality, which probably may be related to the DPI resolution. Otherwise, I suggest approving the paper since we need more work in this area.

Is the work clearly and accurately presented and does it cite the current literature?

Yes

If applicable, is the statistical analysis and its interpretation appropriate?

Yes

Are all the source data underlying the results available to ensure full reproducibility?

Yes

Is the study design appropriate and is the work technically sound?

Yes

Are the conclusions drawn adequately supported by the results?

Yes

Are sufficient details of methods and analysis provided to allow replication by others?

Yes

Reviewer Expertise:

Privacy-preserving methods for genomics privacy.

I confirm that I have read this submission and believe that I have an appropriate level of expertise to confirm that it is of an acceptable scientific standard.

# Peer review: Reviewer response for version 1

**DOI:** 10.21956/wellcomeopenres.18654.r48298

**Published:** 2022-02-02

**Authors:** Bastian Greshake Tzovaras

**Affiliations:** 1 Université de Paris, Paris, France

This article contributes to existing literature on attitudes towards genetic/medical data sharing by presenting the results of the Italian branch of the international *Your DNA, Your Say *study and giving an overview of the attitudes towards the sharing of DNA & medical information among the Italian's public.

 The article itself is well written and presents its arguments clearly. The main limitation I see with respect to presenting the results are within Table 3 & 4. For these, the sizes and focus on only giving percentages makes them hard to interpret the data easily. It could be worthwhile to split these large tables up into more manageable chunks while adding the raw counts of survey answers to allow a better contextualization of the percentages.

 On a methodological level I concur with the review of Ellen Wright Clayton regarding the statistical significance: It would be of value to clearly state how many statistical tests were performed overall, as the number given seems rather high based on the p-values reported in the main text. Correspondingly, and adjustment the significance levels for the multiple testing might be worth consideration.

Is the work clearly and accurately presented and does it cite the current literature?

Yes

If applicable, is the statistical analysis and its interpretation appropriate?

Partly

Are all the source data underlying the results available to ensure full reproducibility?

Yes

Is the study design appropriate and is the work technically sound?

Yes

Are the conclusions drawn adequately supported by the results?

Partly

Are sufficient details of methods and analysis provided to allow replication by others?

Yes

Reviewer Expertise:

Direct-To-Consumer genetic testing and its societal implications.

I confirm that I have read this submission and believe that I have an appropriate level of expertise to confirm that it is of an acceptable scientific standard, however I have significant reservations, as outlined above.

# Peer review: Reviewer response for version 1

**DOI:** 10.21956/wellcomeopenres.18654.r45246

**Published:** 2021-08-04

**Authors:** Ellen Wright Clayton

**Affiliations:** 1 Center for Biomedical Ethics and Society, Vanderbilt University Medical Center, Nashville, TN, USA

This article adds to the wealth of research from about the world about attitudes regarding data sharing. The authors would do well to cite some of the recent systematic reviews of the empirical data on this topic. see, e.g., Kalkman S, van Delden J, Banerjee A, *et al.* (2019) ^1^, as well as articles from Italy Colombo C, Roberto A, Krleza-Jeric K, *et al. *(2019) ^2^. These authors' findings largely align with those reported by others, which is reassuring.

 My biggest reservation is how the data are presented. The tables should present the raw numbers in each box instead of or in addition to the percentages. More important, both the tables and the figures need to identify which differences are statistically significant. At present, these are buried in the text where it is hard to dig them out. In addition, given how many comparisons they did, they should be more careful about claiming significance at 0.05, and there are a lot of percentages given with no comment about significance at all.

 Thanks for the chance to read this.

Is the work clearly and accurately presented and does it cite the current literature?

Partly

If applicable, is the statistical analysis and its interpretation appropriate?

Partly

Are all the source data underlying the results available to ensure full reproducibility?

Yes

Is the study design appropriate and is the work technically sound?

Yes

Are the conclusions drawn adequately supported by the results?

Partly

Are sufficient details of methods and analysis provided to allow replication by others?

Yes

Reviewer Expertise:

Ethical legal and social implications of genomics research

I confirm that I have read this submission and believe that I have an appropriate level of expertise to confirm that it is of an acceptable scientific standard, however I have significant reservations, as outlined above.

## References

[1] : Patients' and public views and attitudes towards the sharing of health data for research: a narrative review of the empirical evidence. *J Med Ethics* .2019; 10.1136/medethics-2019-105651 10.1136/medethics-2019-105651 31719155PMC8717474

[2] : Sharing individual participant data from clinical studies: a cross-sectional online survey among Italian patient and citizen groups. *BMJ Open* .2019;9(2) : 10.1136/bmjopen-2018-024863 10.1136/bmjopen-2018-024863 PMC637754530782920
